# Understanding colossal barocaloric effects in plastic crystals

**DOI:** 10.1038/s41467-020-18043-1

**Published:** 2020-08-21

**Authors:** F. B. Li, M. Li, X. Xu, Z. C. Yang, H. Xu, C. K. Jia, K. Li, J. He, B. Li, Hui Wang

**Affiliations:** 1 School of Physics and Electronics, Hunan Key Laboratory of Super Microstructure and Ultrafast Process, State Key Laboratory of Powder Metallurgy, Central South University, Changsha, 410083 China; 2 College of Materials Science and Engineering, Changsha University of Science & Technology, Changsha, 410114 China; 3 Center for High Pressure Science and Technology Advanced Research, Beijing, 10000 China; 4 Shenyang National Laboratory (SYNL) for Materials Science, Institute of Metal Research, Chinese Academy of Sciences, Shenyang, Liaoning 110016 China

**Keywords:** Energy science and technology, Condensed-matter physics

## Abstract

Plastic crystal neopentylglycol (NPG) exhibits colossal barocaloric effects (BCEs) with record-high entropy changes, offering exciting prospects for the field of solid-state cooling through the application of moderate pressures. Here, we show that the intermolecular hydrogen bond plays a key role in the orientational order of NPG molecules, while its broken due to thermal perturbation prominently weakens the activation barrier of orientational disorder. The analysis of hydrogen bond strength, rotational entropy free energy and entropy changes provides insightful understanding of BCEs in order-disorder transition. External pressure reduce the hydsrogen bond length and enhance the activation barrier of orientational disorder, which serves as a route of varying intermolecular interaction to tune the order-disorder transition. Our work provides atomic-scale insights on the orientational order-disorder transition of NPG as the prototypical plastic crystal with BCEs, which is helpful to achieve superior caloric materials by molecular designing in the near future.

## Abstract (web summary)

Colossal barocaloric effects with high entropy changes is reported in plastic crystal neopentylglycol, while microscopic mechanism needs to be further explored. Here, the authors show hydrogen bond related reorientational dynamics of neopentylglycol and provide insights in order-disorder transition.

## Introduction

Rising demand for cooling is widely expected due to global warming caused by climate change, economic growth, and urbanization. Refrigeration, air conditioning, and heat-pump equipment are already widely used throughout the economy by consuming about 25–30% of the global electric energy^1^. However, most of the cooling process involves conventional vapor compression of hazardous gases, and their working materials and power-supplying stations are subjected to the growing environmental issues such as refrigerant leakage, CO_2_ emissions, etc.^2,3^. Meanwhile, the scalability of current-cooling technologies has been severely restricted, which cannot meet the needs of developing faster microchips and more portable electronic devices in Post-Moore’s law era. Solid-state cooling offers an elegant solution to all these issues; exploring materials with large caloric effects near room temperature is promising and challenging in modern material science, physics, and refrigeration technology based on solid-state caloric effects, which will provide a way to replace cooling devices based on conventional vapor compression^4,5^.

It is known that caloric effects arise at an order–disorder transition induced by external fields. The magnetocaloric effect (MCE) is a magneto-thermodynamic phenomenon in which a temperature change of a suitable material due to ferromagnetic–paramagnetic transition is caused by exposing it to a changing magnetic field, and extremely large magnetic entropy change has been discovered in Gd_5_(Si_2_Ge_2_)^6,7^. Similarly, the electrocaloric effect (ECE) is a phenomenon in which a material shows a reversible temperature change near ferroelectric–paraelectric transition under an applied electric field, and giant ECE in thin-film PbZr_0.95_Ti_0.05_O_3_ was reported^8,9^. The elastocaloric effect (eCE) occurs when stress is applied or removed on a material and a phase transformation is induced; the material heats up or cools down as a result of the entropy difference between the two coexisting phases^10,11^. Differently, the barocaloric effect (BCE) that refers to the isothermal entropy change or adiabatic temperature change on the application or withdrawal of an external pressure, has been realized in various systems such as shape memory alloys^12^, organic–inorganic hybrid perovskites^13^, superionic compounds^14,15^, and plastic crystals^5,16,17^, making it most universal among these solid-state caloric effects. So far, almost all BCE materials show entropy changes of dozens of joules per kilogram per kelvin^12,14,16^, except that plastic crystals such as neopentylglycol (NPG) possess hundreds of joules per kilogram per kelvin near or around room temperature, and hold out promises for next-generation BCE materials in practical applications^17^.

In this work, we show that the formation of hydrogen bond ladder between hydroxy of adjacent NPG molecule columns restricts the rotation of molecules at low temperature, while its breaking significantly lowers the activation barrier. The hydrogen bond and its correlation to the transition temperature are discussed as compared with other plastic crystals. The calculated activation energy barrier, rotating rate, and entropy changes consist of experimental results. Most vibrational frequencies of the NPG molecule are identified in theoretical calculations and Raman spectroscopy measurements, among which only the O–H stretching modes are significantly weakened and pressure dependent. These results provide fundamental understandings of NPG as prototypical plastic crystals in order–disorder transition.

## Results

### Structural stability and hydrogen bond of NPG

**Fig. 1:**
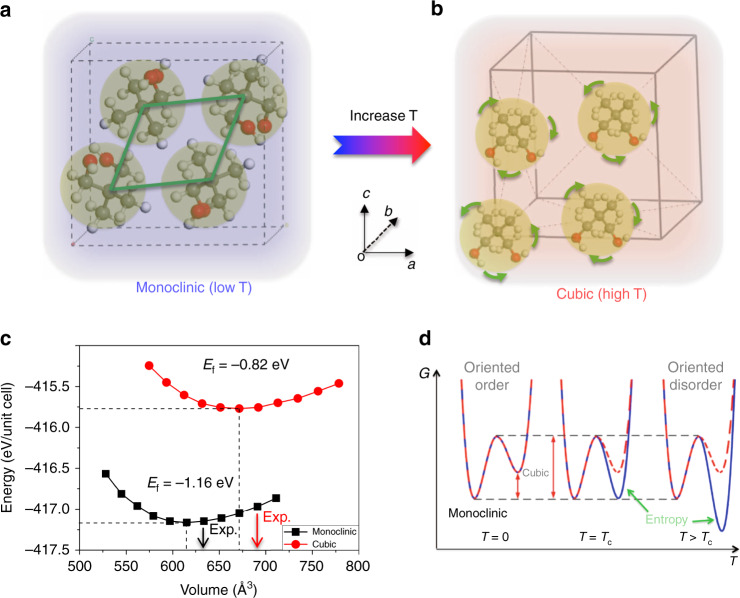
Structure and stability of NPG in different phases. Crystal structure of NPG in (**a**) the monoclinic phase at low temperature and (**b**) cubic phase at high temperature. The light-yellow-shaded circle represents one NPG molecule. The green lines in (**a**) and green arrows in (**b**) demonstrate the stationarity and rotation of the NPG molecule, respectively. **c** Calculated volume dependence of total energy of NPG in monoclinic and cubic phases. *E*_*f*_ represent the formation energy as defined in the following paragraph. Black and red arrows indicate their corresponding experimental values^17^. **d** Schematic diagram indicates the existence of the energy barrier between monoclinic and cubic phases as shown by the red dash line, while the blue solid line marked by green arrows accounts for the entropy contributions to the total Gibbs free energy.

NPG molecule consists of five carbon atoms that form the tetrahedron, in which two carbon atoms are bonded to hydrogen atoms in the methyl group (CH_3_), while the rest two are attached to the hydroxymethyl group (CH_2_OH). Plastic crystals of NPG (chemical formula: C_5_H_12_O_2_) crystallize in the monoclinic phase with space group P2_1_/n below transition temperature (*T*_c_ ~314 K) as shown in Fig. 1a. The ordered structure undergoes monoclinic-to-cubic (or α→γ) phase transition above *T*_c_, where an orientationally disordered face-centered lattice (FCC) formed as evidenced by previous experimental results^17,18^. As shown in Fig. 1b, the random rotation of the entire NPG molecules around their center of mass and their dynamical averaging over time will maintain the FCC symmetry, which is mainly responsible for the energy adsorption induced by thermal agitation.

The equilibrium lattice constant is obtained by calculating the total energy (*E*) of NPG in the monoclinic and cubic phase as a function of cell volume (*V*), as shown in Fig. 1c. Upon increasing the volume, the total energies of the two structures converge and reach their corresponding minimum, at about 615 Å^3^ (*a* = 6.02 Å, *b* = 10.48 Å, and *c* = 9.90 Å, *α* = 90.0, *β* = 100.2, and *γ* = 90.0°) and 672 Å^3^ (*a* = *b* = *c* = 8.76 Å, *α* = *β* = *γ* = 90.0°) for monoclinic and cubic phases, respectively, in good agreement with experimental measurements^17^. As can be seen, monoclinic NPG is about 0.34 eV/molecule energetically stable than the cubic one, identifying the monoclinic phase as the most favorable phase at low temperature. According to the formula of formation energy: *E*_*f*_ = *E*_bulk_ − N*E_molecule_, where *E*_bulk_ and *E*_molecule_ represent the energy of bulk NPG and the isolated NPG molecule, respectively, *N* is number of molecules in the unit cell. The calculated *E*_*f*_ of plastic crystal NPG in monoclinic and cubic phases are −1.16 and −0.82 eV/molecule, respectively, both are energetically stable, as marked in Fig. 1c.

**Fig. 2:**
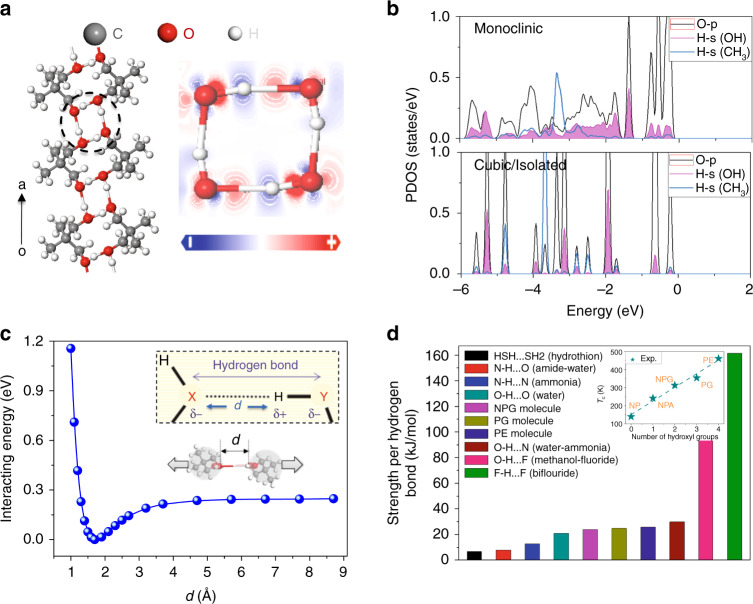
Analysis of hydrogen bond and comparison with other compounds. **a** Left panel shows the hydrogen bond ladders of monoclinic NPG repeating periodically along the a axis, corresponding to the blue rectangle area in Fig. S1a. The right panel shows the charge-density redistribution maps after the formation of intermolecular hydrogen bond at 0.1 e/Å^3^. Blue and red represent the depletion and accumulation of charge, respectively. **b** Calculated projected density of states of NPG in monoclinic and cubic phases. Fermi level locates at zero in energy scale. **c** Calculated interacting energies of between two NPG molecules as sketched by the simulated tensile and compress process from their equilibrium position. The inset demonstrates the hydrogen bond O–H···O in NPG. **d** The strength per hydrogen bond of different types (X–H···Y) in various materials^21–24^, along with the calculated value of NPG, PG, and PE molecules for comparison. The inset shows the plot of phase-transition temperature of NP, NPA, NPG, PG, and PE plastic crystals against the total number of hydroxyl groups per molecule^17,48^.

At low temperature, NPG molecules tend to align with orientational order in monoclinic phase, as demonstrated in Fig. 2a and Supplementary Fig. 1a. The intermolecular hydrogen bond ladders along the *a* axis are formed between O and H atoms of adjacent NPG molecule columns, while the other two sides are separated due to the repulsion of methyl group. The calculated three-dimensional charge density and band structures show that electronic states are mainly localized and disconnected in monoclinic NPG (Supplementary Fig. 1b, c), indicating its insulating nature. However, one should note that electron-wave function starts to overlap where the hydroxyl of the adjacent NPG molecule interacts with each other. The formation of a rectangular hydrogen bond (O–H···O) ladder along the *a* axis between adjacent NPG molecule columns (Fig. 2a) is evidenced by the elongation of bond length of O–H (from its original value 0.973 Å^19^ to ~1.003 Å) and the following observed redshift of O–H stretching mode, as described by Jeffery^20^. As also shown in the right panel of Fig. 2a, the in-plane plot of charge-density difference map reflects the charge redistribution after the O–H···O bond forms, and indicates that weak covalent-like feature developed from charge depletions near H and O atoms that led to charge accumulation between them^21^.

Figure 2b demonstrates that the projected density of states (PDOS) of O and H atoms are largely spread out and resonate in the same energy range from −6.0 to 0 eV in the monoclinic phase, indicating the characteristic of periodical bonding. In the cubic phase, intermolecular hydrogen bond is largely broken and mainly exists in the transient bonding state due to the thermal activated rotation of the NPG molecule. Therefore, the corresponding PDOS in the same energy range shows discrete and separated energy peaks, manifesting the isolated molecular bond nature. In comparison, the PDOS of H atoms of the methyl group demonstrates similar isolated states between −4.0 and −3.0 eV in both phases, in accordance with their unperturbed molecular feature.

At higher temperature, the hydrogen bonds begin to break so that the individual molecules are then free to rotate and vibrate about their centers of mass. Because of these additional vibrational states, plastic crystal NPG is able to store thermal energy without a temperature rise. Above *T*_c_, hydrogen bonds are broken, the NPG molecules are attracted only by van der Waals forces (~0.07 eV/molecule), and the lattice is maintained as FCC symmetry. In addition to factors such as chemical structure details and external pressure, *T*_*c*_ of plastic crystals is related to the hydrogen bond energy and the total number of hydrogen bonds per molecule. It is known that *T*_*c*_ of NPG is ~ 314 K, as it has two hydroxyl groups per molecule to form the hydrogen bond, and the strength of each bond is calculated to be ~0.25 eV as shown in Fig. 2c. Naturally, one can see that as the hydrogen bond enhances and the total number of hydrogen bonds per molecule increases, *T*_*c*_ will rise up accordingly. As evidenced by other plastic crystals such as neopentane (NP, chemical formula: C_5_H_12_), neopentylalcohol (NPA, chemical formula: C_5_H_12_O), pentaglycerine (PG, chemical formula: C_5_H_12_O_3_), and pentaerythritol (PE, chemical formula: C_5_H_12_O_4_)^17^, they show similar structural features to NPG but have zero, one, three, and four hydroxyl groups per molecule to form the hydrogen bond, respectively. Since their hydrogen bond strength is similar (Fig. 2d), the difference of their *T*_c_ is mainly determined by the number of hydrogen bonds per molecule. As demonstrated in the inset of Fig. 2d, *T*_c_ of NP, NPA, NPG, PG, and PE show an approximated linear relationship with the total number of hydroxyl groups per molecule that form the hydrogen bond.

We further analyze and compare hydrogen bond energy in other compounds^21–24^. As shown in Fig. 2d, the hydrogen bond energy of S–H···S (e.g., hydrothion), N–H···O (e.g., amide–water), and N–H···N (e.g., ammonia) is weak and lies below 15 kJ/mol. It is moderate for NPG, PG, and PE molecules with a value of ~25 kJ/mol, which has the same type of hydrogen bond (O–H···O), while it becomes strong and surpasses 30 kJ/mol for O–H···N (e.g., water–ammonia), O–H···F (e.g., methanol–fluoride), and F–H···F (e.g., bifluoride). Since most plastic crystals possess hydrogen-bonded groups (different types of X–H···Y), they serve as a simple rule to achieve caloric materials by choosing the specific hydrogen bond where the order–disorder transition occurs under different temperature. Therefore, these results provide useful guidance for the selection of the proper type of hydrogen bond groups in synthesizing plastic crystals for application.

### Reorientational dynamics in order–disorder transition

**Fig. 3:**
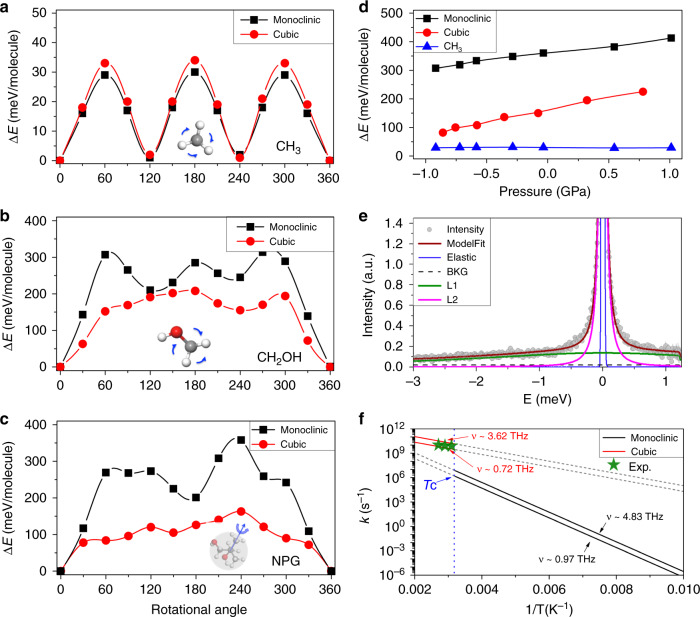
Activation barrier and reorientational dynamics of NPG. Calculated activation energy barrier of rotating of (**a**) CH_3_ group, (**b**) CH_2_OH group, and (**c**) the entire NPG molecule in both monoclinic and cubic phases. **d** Pressure-dependent activation energy barrier of the rotating CH_3_ group and the entire NPG molecule. **e** Inelastic neutron-scattering profiles as a function of energy transfer *E* at 1.65 Å^−1^ ≤ *Q* ≤ 1.75 Å^−1^ at temperature 320 K. The experimental data were reproduced based on a combination of a constant background (BKG), a resolution-convoluted delta function (elastic), and two Lorentzian functions (L1 and L2). **f** Calculated temperature dependence of rotating rate (in log scale) for the NPG molecule in monoclinic and cubic phases. Green star indicates their corresponding experimental values obtained from neutron-scattering data at different temperatures above *T*_*c*_, as shown in Supplementary Fig. 2a, b. The two lines at each phase show the value by changing attempt frequency; gray dash lines are guided for your eyes. The simulated temperature is from 100 to 500 K, and the vertical blue line indicates the critical temperature of phase transition.

In the context of energy landscape, the monoclinic-to-cubic phase transition does not occur spontaneously, which requires thermally excited process to overcome the activation barrier between these two phases as demonstrated in Fig. 1e. To illustrate that, we sequentially calculate the rotational energy barriers of CH_3_, CH_2_OH group, and the entire NPG molecule in monoclinic and cubic phases. The rotational energy barrier of the CH_3_ group first increases to maximum at 60° and then decreases down to minimum at 120°, and repeats this process until it reaches its initial position 360°, indicating the characteristic of threefold symmetry (C_3_). The barrier is ~30 meV in the monoclinic phase, which is similar to that in the cubic phase (Fig. 3a). In contrast, owing to the asymmetric nature, the rotational energy barrier of the CH_2_OH group in the monoclinic phase increases rapidly to its maximum at 60° and oscillates near or around 300 meV, then decreases down to zero after the rotational angle surpasses 300° that is substantially lowered in the cubic phase as shown in Fig. 3b.

The rotational energy barrier of the entire NPG molecule follows the trend of the CH_2_OH group in two phases, with its maximum up to 360 meV in the monoclinic phase (Fig. 3c) during the rotational process. It is worth noting that the significant high-energy barrier of the rotating NPG molecule in the monoclinic phase is mainly related to the intermolecular hydrogen bond (demonstrated in Fig. 2), which impedes it from rotating away. While the hydrogen bond is significantly broken in the cubic phase as evidenced by the reduction of the barrier below 150 meV (Fig. 3c). Furthermore, Fig. 3d shows that external pressure does not affect the activation barrier of CH_3_ groups as they are relatively isolated; in contrast, it enhances the activation barrier of the entire NPG molecule in both monoclinic and cubic phases with compressive pressure.

Based on the activation barrier, an estimation of rotating rate of the NPG molecule can be obtained based on the canonical transition-state theory. According to the Arrhenius equation^25,26^, the transition rate coefficient can be obtained by equation

1 $$k = ve^{ - \frac{{{\Delta}E}}{{k_BT}}},$$

where *ν* is the attempt frequency, Δ*E* is the activation barrier for rotation, *k*_*B*_ is Boltzmann’s constant, and *T* is temperature in kelvin. In general, the attempt frequency can be estimated through analysis of the frequency of related modes^27^. Previous inelastic neutron-scattering results indicate that these modes at low temperature typically locate below 20 meV (~4.83 THz), while they soften substantially (as large as 25%) above phase transition^17^. We then set attempt frequencies in a range of [0.97–4.83] THz and [0.72–3.62] THz for low and high temperature, respectively, and calculate the temperature-dependent rotating rate of the NPG molecule. As seen in Fig. 3f, the rotating rate at low temperature (<100 K) is negligible (~10^−6^ s^−1^), indicating the orientation-ordered nature in the timescales of neutron-scattering processes; as temperature rises, the rotating rate of the NPG molecule increases accordingly. Above *T*_c_, it changes significantly and increases by 4–5 orders of magnitude, ascribed to the hydrogen bond broken and the resultant reduction of the activation energy barrier when orientational order–disorder transition occurs. Although the process of order–disorder transition is complicated, the rough estimation based on Arrhenius model indicates that the rotational process should be completed in ~100 ps, in accordance with the relaxation time obtained from our inelastic neutron-scattering measurement near room temperature (Fig. 3e, f; Supplementary Fig. 2a, b).

The transition between monoclinic and cubic phases in plastic crystals of NPG can be cast into the variation of total Gibbs free energy^28,29^, which consists of changes in the internal energy (Δ*U*) and rotational Helmholtz free energy [−*T*Δ*S*, where S is the entropy of the system] and the energy change incorporated with the change in volume at a given pressure (*P*Δ*V*). Our experimental results suggest a volume change of ~10% near *T*_*c*_ at ambient pressure; we estimate *P*Δ*V* work of ~0.04 meV/unit cell (*P*Δ*V*_work_ = *P*_external_ × Δ*V*, where *P*_external_ is the barometric pressure 1 atm, Δ*V* is the volume change)^17^. Thus, the prominent difference in Gibbs free energy between the two phases is dominated by the rotational entropy of NPG molecules^30^.

Our previous results show that NPG molecules have many degenerated states during the random rotational process above temperature of phase transition^17^, so it is reasonable to model the molecule as a free 3D rigid rotor in the disordered phase. The validity and reliability has already been successfully used and verified in systems with similar properties such that the molecule undergoes order–disorder transition^31^. According to statistical mechanics, the partition function of a freely rotating molecule is given by

2 $$Q_{\rm{rot}}(T) = \frac{{8{\uppi}^2}}{{\sigma h^3}}\left( {2{\uppi}k_BT} \right)^{\frac{3}{2}}\left( {I_xI_yI_z} \right)^{\frac{1}{2}}$$

from which the rotational entropy (*S*_rot_) of a nonlinear molecule consisting of multiple atoms can be derived as follows:

3 $$S_{\rm{rot}}(T) = Rln\frac{{\left( {I_xI_yI_z} \right)^{1/2}}}{\sigma } + \frac{3}{2}{\rm{RT}} + \left[ {\frac{3}{2}R + \frac{R}{2}ln\pi \left( {\frac{{8{\uppi}^2k_B}}{{h^2}}} \right)^3} \right]$$

where *R* is gas constant, *h* is Plank constant, *T* is temperature in kelvin, *σ* is the number of orientations of the molecule that interchange only identical atoms, and *I*_*x*,_ *I*_*y*,_ *I*_*z*_ are the principal moments of inertia of the NPG molecule, which are calculated to be 178.884, 207.785, and 271.256 uÅ^2^ (the unified atomic mass unit, u, is 1.66 × 10^−27^ kg). Because the rotational entropy contribution is negligible in the monoclinic phase due to the large activation barrier, the Helmholtz free energy difference between the two phases becomes Δ*F*(*T*) ~ −*TS*_*rot*_, which is about −383 meV at *T*_*c*_ = 314 K. Thus, this entropy contribution significantly lowers the Gibbs free energy of the cubic phase well below the monoclinic phase, as illustrated in Fig. 1d.

As molecular rotation leads to orientational disorder, the entropy changes at the order–disorder transition can be estimated by counting the accessible microscopic configurations of the molecules^32^. In the disordered state with FCC symmetry, NPG molecules have many degenerated states during the random rotational process. As reported in previous work^32^, molecules of tetrahedral symmetry assume two orientations in which the common molecular and lattice symmetry elements belong to the *T*_*d*_ point group and an additional eight orientations of C_3v_. So, NPG molecules are randomly distributed among the 10 orientations of the *T*_*d*_ and C_3v_ sets. In addition, there are six orientations of the two CH_2_OH groups appearing on the four corners of the tetrahedron. For each CH_2_OH group, there are three orientations with respect to the tetrahedron. As a result, the total number of molecular orientations is 10 × 6 × 3 = 180, which leads to *R*ln(180) = 415 J kg^−1^ K^−1^ at temperature 314 K according to Boltzmann’s equation. It is worth noting that only limited microscopic configurations are considered under FCC symmetry; the value is already larger than the experimentally observed entropy changes of NPG molecules^17^. This could be ascribed to the fact that not all the orientations are fully activated at the temperature of phase transition, and some orientations might be restricted by geometry. However, using configurational entropy to calculate the entropy changes sheds light on understanding the orientational ordered–disordered phase transition of NPG molecules.

### Characterization of vibrational properties of NPG

**Fig. 4:**
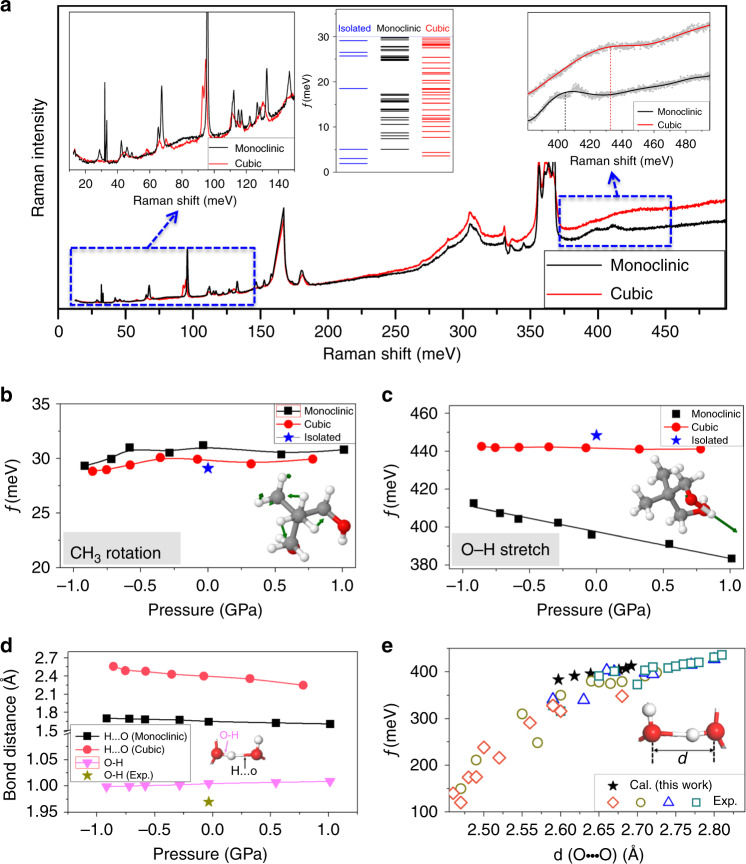
Pressure-dependent vibrational properties and hydrogen bond length of NPG. **a** Raman spectroscopy measurements of NPG plastic crystal in monoclinic and cubic phases with energy range between 10 and 480 meV. The insets demonstrate the low-energy (from 20 to 140 meV) and high-energy range (from 390 to 490 meV), respectively. The pressure-dependent (**b**) CH_3_ rotation and (**c**) O–H stretching modes of NPG molecules in isolated, monoclinic, and cubic phases; the insets visualize the corresponding mode. Negative and positive values in the horizontal axis represent tensile and compressive pressure, respectively. **d** Bond length of the covalent bond O–H and hydrogen bond O–H···O of NPG molecules at different pressure, along with experimental O–H covalent bond length^19^. The hydrogen bond O–H···O of NPG molecules in the cubic phase is estimated by the average of all O–H···O in the unit cell. **e** Calculated stretching frequencies of O–H bond against O···O distances in the hydrogen bond (O–H···O) of NPG, along with experimental infrared spectroscopic results^20^ (diamonds: combination of acid and complementary base; circles: resonance-assisted hydrogen bond; triangles: σ-cooperative hydrogen bonds; squares: isolated hydrogen bonds).

The order–disorder transition-induced changes of vibrational properties of NPG molecules can be obtained through Raman scattering measurement. As shown in Fig. 4a, a series of peaks corresponding to different modes show up as the energy of the laser increases from 10 to 480 meV. The orientational disorder of NPG plastic crystals maintains FCC symmetry after dynamical averaging over time, leading to the fact that some peaks are broadened or disappeared in the cubic phase, as compared with those in the monoclinic phase (left inset in Fig. 4a for the low-frequency range).

To identify these modes and understand how they are affected by external pressure, density functional perturbation theory (DFPT) calculations are performed. The broadened feature in the cubic phase is confirmed by comparing the vibrational frequencies of NPG in isolated and monoclinic phases (inset in Fig. 4a). The low-frequency rotational mode of the CH_3_ group is found at around 30 meV in both monoclinic and cubic phases, close to the value of the isolated NPG molecule as shown in Fig. 4b. High-frequency modes, such as CH_3_ stretching, C–H swinging, and C–H stretching mode, are identified at about 365, 175, and 375 meV, respectively, as shown in Supplementary Fig. 3a–c. Interestingly, these modes are almost unaffected at different external pressure in a range of [−1.0 to 1.0] GPa in both phases, indicating the nature of their intrinsic attributes.

Importantly, we observe distinct results for O–H stretching mode in monoclinic and cubic phases. As shown in Fig. 4c, the vibrational frequency of O–H stretching mode in the cubic phase locates near 440 meV (similar to the value of the isolated NPG molecule, 445 meV), while it decreases down to ~400 meV in the monoclinic phase, in good agreement with Raman scattering results (the right inset of Fig. 4a). The O–H stretching mode shows almost linear decrement, and its bond length is slightly increased as external pressure changes from ~−1.0 GPa to ~+1.0 GPa (Fig. 4c, d). Due to the giant compressibility of NPG plastic crystals, external pressure shrinks the space between molecules and decreases the hydrogen bond length (O–H···O) (Fig. 4d), which further enhances the activation energy barrier and restricts the rotation of NPG molecules (Fig. 3d). Therefore, external pressure is able to induce large changes in the lattice dynamics, and plays a key role in the orientational order–disorder transition of plastic crystal NPG. It is also worth noting that the vibrational frequency of O–H in O–H···O hydrogen bonds under different pressure is correlated with the O···O distances, matching well with previous established data (Fig. 4e)^20,33^.

## Discussion

Most of the atomic degrees of freedom in plastic crystals are disordered, and only the center of mass of molecules is ordered to support the lattice through vdW interactions, making them the most disordered solids and their transitions to ordered phases perhaps physically define the maxima of entropy changes that are one order of magnitude larger than previous barocaloric materials^5,16^. Moreover, molecules in plastic crystals may possess electric dipole and can respond to external electric bias. In view of this, applying multiple-field cycle combining external pressure and the electric field to tune the barocaloric properties of plastic crystals is promising for practical cooling applications^34^.

Given that the hydrogen bond is ubiquitous in nature and influences the structure, stability, dynamics, and orientation of molecules, the present study provides atomic-scale insights and extensive understandings on the orientational order–disorder transition of NPG as prototypical plastic crystals with BCEs through a systematic theoretical study along with experimental support, which may also ignite the immediate interests in diverse disciplines, including physics, chemistry, materials sciences, energy, biology, and solid-state refrigeration technology.

## Methods

### Theoretical calculations

Density functional calculations were performed with the Vienna Ab-initio Simulation Package (VASP)^35,36^. The generalized gradient approximation with the Perdew–Burke–Ernzerhof functional was used for the description of the exchange-correlation interaction among electrons^37^. We treated C–2s2p, H–1s, and O–2s2p as valence states and adopted the projector-augmented wave (PAW) pseudopotentials to represent the effect of their ionic cores^38,39^. In general, it is believed that the weakly interacted systems can be considerably improved by using nonlocal van der Waals correction^40^, which has been successfully used in various systems^41,42^. By adopting the Γ-centered Monkhorst–Pack method^43^, we sampled the Brillouin zone (BZ) with a k-mesh density of 2*π* × 0.03 Å^−1^ for structure relaxation and total energy calculation. The energy cutoff for the plane-wave expansion was set as 500 eV to converge the computed ground-state properties, in good accordance with previous studies related to carbon, hydrogen, and oxygen elements^41,42,44,45^. Structures were optimized with a criterion that the atomic force on each atom becomes weaker than 0.01 eV/Å, and the energy convergence is better than 10^−6^ eV. DFPT is carried out within the density functional framework to calculate the vibrational properties of crystalline materials^46^.

### Experimental measurements

The Raman spectroscopy was measured in a Renishaw inVia system equipped with a 488-nm laser source. A panoramic diamond anvil cell was used for sealing the sample, and a metal ceramic heating ring was mounted around the diamond for heating. The pressure was calibrated by Ruby fluorescence, and the temperature was measured by touching a K-type thermal couple to the back culet of the diamond anvils. The inelastic neutron-scattering experiment was performed using the cold-neutron disk chopper spectrometer AMATERAS at J-PARC in Japan^47^. The experimental details and spectral fitting were described in previous work^17^.

## Supplementary information

Supplementary Information

## Data Availability

All relevant data are presented via this publication and supplementary information.
