# Supplementary material for: Understanding colossal barocaloric effects in plastic crystals
Source: Nat Commun. 2020 Aug 21;11:4190. doi: 10.1038/s41467-020-18043-1 (PMC7442785; doi:10.1038/s41467-020-18043-1)
Supplement: Supplementary file 1 — Supplementary Information [file 41467_2020_18043_MOESM1_ESM.pdf]

Supplementary Information on

Understanding Colossal Barocaloric Effects in Plastic Crystals

Li et al.

Content

Supplementary Figure 1. Atomic configuration and visualization of hydrogen bond ladder of NPG in monoclinic phase, electronic band structures under different external pressures.

Supplementary Figure 2. Incoherent neutron scattering profiles as a function of energy E at different temperature.

Supplementary Figure 3. The pressure dependent vibrational properties of NPG.

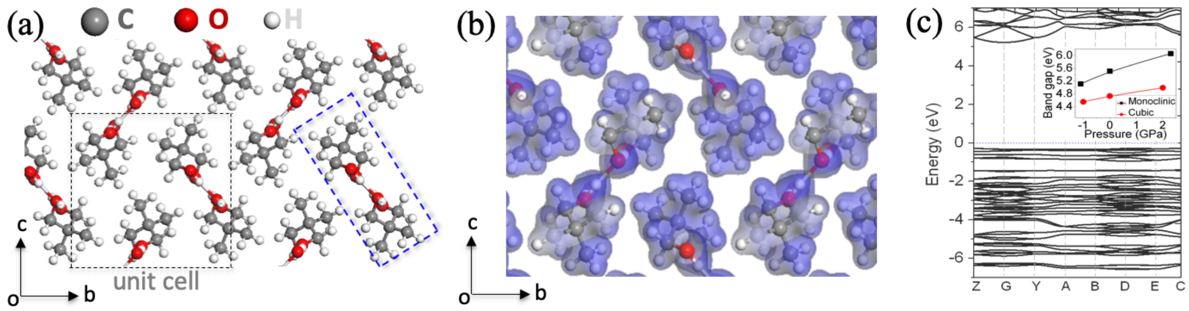

Supplementary Fig. 1 (color online) (a) Atomic configuration of NPG in monoclinic phase, atom positions are projected on the a axis. The black dashed rectangle indicates the unit cell and the blue dashed rectangle shows the hydrogen-bond ladders. (b) Calculated three-dimensional charge density isosurface of monoclinic NPG. The charge density is largely separated between NPG molecules except that the area near hydroxyl where electron wave function overlaps. (c) Calculated electronic band structures of NPG plastic crystal. Inset shows the pressure dependence of band gap of NPG in monoclinic and cubic phase.

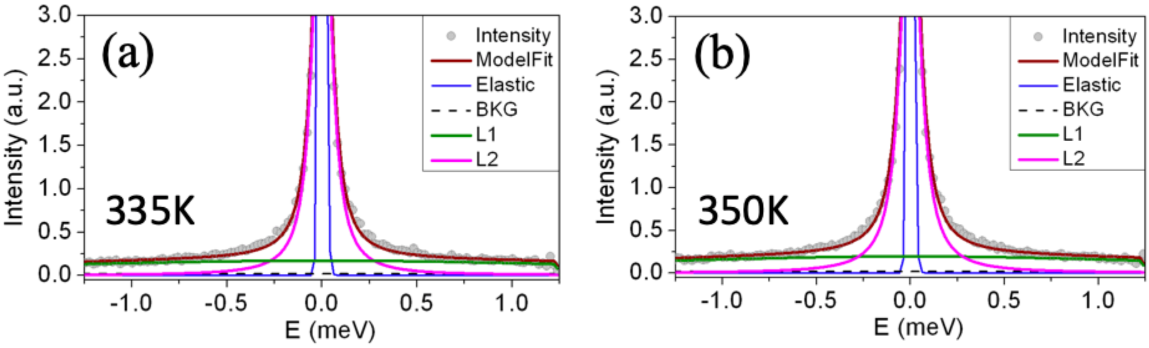

Supplementary Fig. 2 (color online) Incoherent neutron scattering profiles as a function of energy E at 1.65 Å^{-1}≤ Q ≤ 1.75Å^{-1} at temperature (a) 335K and (b) 350 K, respectively.

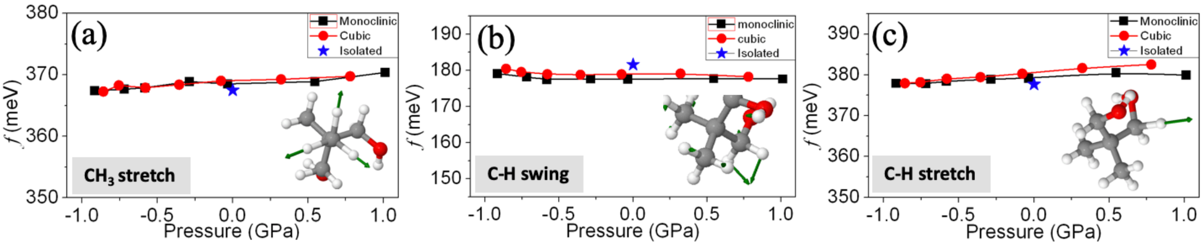

Supplementary Fig. 3 (color online) The pressure dependent of (a) CH_3 stretch, (b) C-H swing and (c) C-H stretch modes of NPG molecules in isolated, monoclinic and cubic phase, the insets visualize the corresponding mode. Negative and positive values in horizontal axis represent tensile and compressive pressure, respectively.
